# Association between interleukin 1β and interleukin 10 concentrations: a cross-sectional study in young adolescents in Taiwan

**DOI:** 10.1186/1471-2431-13-123

**Published:** 2013-08-14

**Authors:** Jung-Su Chang, Chun-Chao Chang, Eve Yiwen Chien, Sean S-H Lin, Tsai Cheng-Shiuan, Chyi-Huey Bai, Kuo-Ching Chao

**Affiliations:** 1 School of Nutrition and Health Sciences, College of Public Health and Nutrition, Taipei Medical University, Taipei, Taiwan R.O.C; 2 Division of Gastroenterology and Hepatology, Department of Internal Medicine, Taipei Medical University Hospital, Taipei, Taiwan R.O.C; 3 Department of Internal Medicine, School of Medicine, College of medicine, Taipei Medical University, Taipei, 110, Taiwan R.O.C; 4 School of Public Health, Taipei Medical University, Taipei, Taiwan R.O.C; 5 Department of Public Health, College of Medicine, Taipei Medical University, Taipei, Taiwan R.O.C

**Keywords:** IL10, IL1β, Overweight and obese adolescents, Taiwan

## Abstract

**Background:**

In adults, low circulating interleukin 10 (IL10) has been associated with obesity and type 2 diabetes. However, studies investigating IL10 in overweight and obese children have yielded conflicting results. The aim of this study was to investigate factors associated with serum IL10 concentration in young Chinese adolescents.

**Methods:**

Young adolescents (n=325) ages 13.33±1.10 years were recruited into the cross-sectional study from 2010 to 2011. Parameters of obesity, individual components of MetS, iron status and serum IL10 were evaluated.

**Results:**

Compared with their normal weight counterparts, overweight adolescents had lower serum IL10 but higher TNFα, nitric oxide (NO) and IL1β concentrations (all p<0.05). Obese adolescents had increased IL1β but decreased hepcidin concentration compared with normal weight (p<0.01 and p<0.05; respectively). A strong inverse relationship (p<0.0001) was found between IL10 and pro-inflammatory cytokines (TNFα and IL1β). Multivariate linear regression analysis showed serum IL1β was significantly correlated with IL10 (β=−0.156, p<0.0001). When overweight and obese adolescents were assessed separately from normal weight, only IL1β was inversely associated with serum IL10 (β=−0.231, p=0.0009). The association between IL10 and IL1β was weaker in adolescents with normal weight (β=−0.157, p=0.0002), after adjusting for gender, TNFα, IFNγ and NO.

**Conclusions:**

Our study confirmed that low IL10 concentration is associated with overweight and obesity in young adolescents. We also demonstrated for the first time that pro-inflammatory cytokine IL1β is independently associated with IL10. A decline in IL10 concentration in overweight and obese adolescents may further contribute to the IL1β-mediated inflammatory environment associated with obesity.

## Background

Interleukin 10 (IL10) plays a central role in regulating immune response and limiting inflammation. IL10 suppresses inflammation through various mechanisms including inhibition of the synthesis of pro-inflammatory cytokines such as IL12 and TNFα via suppression of p65 NF-kB and c-rel activity in macrophages [1]. IL-10 is also important in down-regulation of the release of reactive oxygen species and nitrogen intermediates; regulation of antigen presentation capacity and immune tolerance [2]; and suppression of proliferative and cytotoxic T cell responses [3].

There is growing evidence linking IL10 to obesity [4], metabolic syndrome (MetS) and cardiovascular disease [5]. In adults, low circulating IL10 has been associated with obesity [6], cardiovascular disease [7-9] and type 2 diabetes [5,10-12]. Clinical significance of circulating IL10 concentrations have been demonstrated in acute coronary syndrome [8,9] and insulin resistance [5,10-12]. The balance of pro-and anti-inflammatory cytokines is an important determinant of atherosclerotic plaque instability. IL10 expression has been found within human atherosclerotic plaques [13]. Smith *et al.* studied 95 patients with angina and related coronary artery disease and observed significantly lower serum IL10 concentrations in patients with unstable angina compared with those with stable angina [9]. Heeeschen *et al.* investigated the prognostic impact of C-reactive protein (CRP) and IL10 in patients with acute coronary syndrome and reported elevated IL10 concentrations were strongly associated with prognosis of acute coronary syndromes [8]. The predictive value of IL10 was independent of myocardial necrosis but significantly interacted with CRP concentration, suggesting the importance of the balance between pro-inflammatory and anti-inflammatory cytokines as a major determinant of patient’s outcome in acute coronary syndromes [8].

IL10 also plays a protective role against the development of insulin resistance. Plasma IL10 concentrations were positively correlated with insulin sensitivity in young healthy adults (r=0.37, p=0.00023) [11]. Forte *et al.* performed the grade-of-membership analysis in 490 type 2 diabetic and 349 control subjects of Italian Caucasians [14]. The authors reported 74.4% of subjects negative for IL-10 -824 T allele were diabetic patients characterized by vascular damages, suggesting that IL10 -597A/-824 T/-1087A negative subjects are more prone to the major type 2 diabetic vascular damages [14].

Studies investigating IL10 in children and young adolescents with obesity have yielded conflicting results [15-18]. According to a study by Gozal and colleagues, obese children with obstructive sleep apnea had low plasma IL10 concentration [17]. In a study including 70 severely obese and 30 normal weight children aged 11.46±3.42 years old, Calcaterra and colleagues reported that serum IL10 concentrations were higher in severely obese children compared to normal weight [16]. Tam *et al.* reported no differences in IL10 concentration between normal and overweight children at 8 years old; however, at 15 years old, serum IL10 was elevated in overweight and obese girls when compared to normal weight girls of same age [18]. These data suggest that effects of obesity on IL10 serum concentration vary with age, sex and obesity-related complications. Additionally, the possible association between IL10 and iron status has also been highlighted in some studies. For instance, patients receiving high doses of IL10 developed hyperferritinemia [19] whereas iron supplementation in IL10- knock out mouse increased pro-inflammatory cytokine production in high iron diet group compared with chow diet [20].

In adults, IL10 plays a protective role against the development of obesity-related declines in health. However, the effect of obesity on IL10 concentration in children is not clear. We therefore measured IL10 concentration and investigated the factors associated with IL10 homeostasis in young adolescents. The variables analyzed included: 1) anthropometry; 2) individual components of metabolic syndrome; 3) pro-inflammatory and oxidative stress markers; and 4) iron parameters.

## Methods

### Study design and participants

This cross-sectional study involved young adolescents from 2 junior high schools located in Taipei and New Taipei City, Taiwan. From September 2010 to November 2011, 340 subjects (ages 13.33±1.10 years, 182 boys and 158 girls) were enrolled in the study. Exclusion criteria were as follows: individuals with missing data for clinical biochemistry and anthropometry (n=14) and individuals with abnormal serum ferritin concentration (>500 ng/ml) (n=1). A total of 325 adolescents were included in the analysis. Informed parental consent was obtained for enrollment into the study. The study was approved by the Research Ethics Committee of Taipei Medical University (201204011).

### Data collection

Data was collected from the subjects by the same medical staff from the Taipei Medical University Hospital using the same methods and tools. Children were advised not to drink or eat after midnight or exercise 24 hours prior to data collection. On the morning of the study, fasted children who were free of medical conditions were admitted to the school-based health center. Body weight, height, waist circumference,% body fat and blood pressure measurements were obtained by standard methods as described elsewhere [21]. Waist circumference was measured at the midpoint between the lower edge of the rib cage and the top of the iliac crest [22]. Age-sex specific cut off point for body mass index (BMI) was used to define overweight and obesity in adolescent according to guidelines of the Department of Health in Taiwan [23,24]. BMI was calculated as mass (Kg)/[height(m)]^2^. Subjects with BMI greater than 85th percentile of age-sex-specific value were grouped as overweight while those with BMI greater than 95th percentile were classified as obese. Percentage of body fat was estimated by bioelectrical impedance method (Omron Body Fat Analyzer HBF-306).

Metabolic syndrome (MetS) was defined based on the modified International Diabetes Federation (IDF) criteria for 10-<16 years old children and adolescents [25]. Individuals with the presence of ≧3 criteria listed below were classified as MetS: (1) waist circumference: ≥90 cm for boys and ≥80 cm for girls; (2) triglyceride (TG): ≥150 mg/dL; (3) fasting plasma glucose (FPG): ≥100 mg/dL; (4) high density lipoprotein cholesterol (HDL): <40 mg/dL for boys and < 50 mg/dL for girls; and (5) systolic blood pressure (SBP)≥130 mmHg or diastolic blood pressure (DBP)≥85 mmHg.

### Blood biochemistry examination

Fasting blood samples were collected in vacuum tubes containing either EDTA or lithium heparin. All blood samples were separated into red blood cells and serum and stored at −80°C until analysis. Total cholesterol (TC), LDL, HDL and TG were determined by an autoanalyzer (Hitachi 737, USA). Fasting plasma glucose concentration was detected using a glucose oxidase method (YSI 203 glucose analyzer, Yellow Springs Instruments, Yellow Springs, OH). Serum ferritin was measured using a commercially available electrochemiluminescence immunoassay and was quantitated by the Roche Modular P800. Serum iron and total iron binding capacity (TIBC) were measured by ferrozine-based colorimetric method. Percentage transferrin saturation (% TS) was calculated by serum iron/TIBC × 100%. Serum hepcidin was assessed by an enzyme-linked immunosorbent assay (DRG International Inc; Marburg). The assay dynamic range is between 2.5 ng/ml-140 ng/ml. Definitions of abnormal biochemistry blood lipid, glucose profiles [26] and iron status [27] are described elsewhere. Cytokines concentrations (IL1β, TNFα, IL10, IFNγ) were determined by Enzyme-Linked Immunosorbent Assay kit (Procarta Cytokine Assay Kit; Affymetrix, Inc., USA) according to the manufacturer’s instructions. Nitric oxide (NO) concentration in the serum was determined by Griess reagent system. The coefficients of variation (CV) for normal weight and overweight/obese adolescents were: IL1β (36.1% v.s. 36.4%), TNFα (41.2% v.s. 48.9%), IL10 (26.9% v.s. 28.9%), IFNγ (36.4% v.s 30.8%) and NO (89.5% v.s. 86.3%).

### Statistical analyses

Statistical analyses were performed using the Statistical Analysis Systems software (SAS version 9.22; SAS Institute, Inc). Normally distributed data were presented as means ± standard error of mean (SEM). Differences between groups were analyzed by the unpaired t-test. Chi-square or Fisher's Exact Test were used for comparison of proportions. Variables not normally distributed were natural log-transformed to achieve a normal distribution but untransformed values were used for reporting results. The association between serum IL10 concentration and clinical and blood biochemistry parameters were assessed using Pearson’s correlation coefficient. A multivariate linear regression model was used to examine the relationship between the dependent variable (serum IL10) and potential variables. The P value was p<0.05 except for the analysis in Table 1 where a value of p<0.0023 was used based on Bonferroni correction for multi-comparisons.

**Table 1:** Clinical and biochemical characteristics of young adolescents according to IL10 tertiles (n=325, boys=175 and girls=150)

**Variables**^**#**^	T1^*^		T2^*^		T3^*^		***P-trend***
BMI (kg/m^2^)	20.97	±4.3	20.65±	4.3	19.99	±3.2	0.0740
Body fat (%)	22.64	±8.7	21.95±	7.6	20.78	±6.78	0.0818
Waist circumference (cm)	71.21	±11.9	70.55±	10.8	69.16	±9.9	0.1788
Fasting glucose (mg/dL)	90.99	±8.67	91.81±	6.07	92.25	±7.79	0.2240
Fasting serum insulin (μIU/ml)	16.95	±14.2	16.87±	10.3	17.22	±15.8	0.8848
HOMA-IR	3.98	±4.0	3.88±	2.5	4.05	±4.2	0.8940
Sytolic BP (mmHg)	110.89	±11.0	112.33±	8.9	110.75	±8.5	0.9094
Diastolic BP (mmHg)	62.88	±8.2	63.80±	8.7	63.41	±7.7	0.6371
HDL cholesterol (mg/dL)	54.06	±12.3	56.31±	13.4	56.59	±12.67	0.1481
LDL- cholesterol (mg/dL)	89.44	±27.0	91.64±	21.2	90.73	±24.2	0.6998
Triglyceride ((mg/dL))	76.53	±35.6	70.85±	31.2	78.23	±49.6	0.7537
Total cholesterol (mg/dL)	159.9	±30.2	159.63±	24.2	160.75	±30.1	0.8420
Serum iron (ug/dL)	87	±35.8	86.52±	33.1	95.32	±36.0	0.0833
Serum TIBC (ug/dL)	352.1	±44.6	348.56±	47.2	337.01	±37.2	0.0114
Serum ferritin (ng/ml)	57.87	±36.1	55.22±	37.0	55.92	±32.0	0.6844
Transferrin Saturation (%)	25.17	±10.7	25.14±	9.6	28.63	±11.1	0.0167
Hepcidin (ng/ml) ^§^	422.46	±418.7	554.71±	446.7	698.92	±420.7	<0.0001
TNFα (pg/ml) ^§^	24.56	±11.2	18.96±	7.3	18.81	±6.7	<0.0001
IFNγ (pg/ml)	6.72	±1.7	6.34±	2.3	6.24	±2.5	0.1148
NO (uM) ^§^	9.31	±4.3	7.10±	4.2	6.69	±4.3	<0.0001
IL1β (pg/ml) ^§^	1.26	±0.4	1.00±	0.4	0.86	±0.2	<0.0001

^*^IL10 cut-off point: Tertile 1: <8.03 pg/ml, Tertile 2>=8.03 pg/ml <=10.6 pg/ml, Tertile 3>10.6 pg/ml.

^#^Data are presented as means±standard error of mean (SEM) and percent for continuous and categorical variables respectively.

^§^Bonferroni correction p<0.0023.

## Results

### Participant characteristics

A total of 325 children were entered in this study. 231 children were classified as normal body weight while 94 children were diagnosed as overweight or obese. Table 2 shows clinical and biochemical characteristics of study subjects according to nutritional status. The prevalence of overweight was 13.5% (19.67% for boys and 18.30% for girls) and obesity was 15.4% (23.77% for boys and 19.26% for girls). Children with high triglyceride concentration were 5.5% (4.0% for boys and 7.3% for girls) while 21.5% had low HDL (7.43% for boys and 38% for girls). About 12% of children had elevated fasting glucose concentration (15.4% for boys and 8% for girls). Only one male adolescent had fasting plasma glucose concentration >126 mg/dl. Five girls were diagnosed with MetS, which was 1.58% of all the subjects.

**Table 2:** Clinical and biochemical characteristics of children according to nutritional status

	**Normal**		**Overweight**		**Obese**		**Ordinary**
	**Medium**	**IQR**	**Medium**	**IQR**	**Medium**	**IQR**	**P-trend**
Number (boys/girls)	231 (122/109)		44 (24/20)		50 (29/21)		
Height^¤^ (cm)	155.51	7.33	156.62	6.84	159.64^##^	6.55	0.0002
Weight^¤^ (kg)	44.67	6.68	57.38^**^	5.32	70.80^##^	9.59	<0.0001
Waist^¤^ (cm)	64.96	6.20	78.60^**^	5.87	87.07^##^	9.56	<0.0001
Body fat^¤^ (%)	18.57	5.56	26.51^**^	4.52	32.10^##^	7.45	<0.0001
Fasting glucose (mg/dL)	90.00	8.00	91.50	8.50	92.00^#^	11.00	0.0005
Fasting serum insulin	11.37	5.96	16.20^**^	6.19	28.44^##^	19.68	<0.0001
HOMA-IR	2.58	1.40	3.63^**^	1.50	6.50^##^	4.58	<0.0001
SBP (mmHg)	109.00	12.00	111.50	15.50	116.00^##^	11.00	<0.0001
DBP (mmHg)	62.00	11.00	61.00	10.00	66.00^##^	8.00	0.0065
HDL- cholesterol (mg/dL)	58.00	17.00	49.00^**^	14.00	42.50^##^	13.00	<0.0001
LDL- cholesterol (mg/dL)	88.00	29.00	92.50	36.50	88.50	24.00	0.6458
Total cholesterol (mg/dL)	158.00	37.00	160.50	47.00	151.00	33.00	0.1283
Triglyceride (mg/dL)	62.00	32.00	68.00	44.00	78.00^##^	51.00	<0.0001
Serum iron (ug/dL)	86.00	45.00	87.50	39.00	80.00	38.00	0.3875
Serum TIBC (ug/dL)	342.00	58.00	359.00	61.00	343.00	59.00	0.6752
Serum ferritin (ng/dL)	50.00	42.00	40.00	42.50	47.00	34.00	0.4136
Transferritin Saturation (%)	24.90	14.18	25.32	15.65	22.89	11.51	0.5224
Hepcidin (ng/ml)	123.19	22.02	107.26	25.80	120.07^#^	34.35	0.0062
TNFα (pg/ml)	16.69	10.37	21.42^*^	12.30	21.56	11.44	0.1982
IFNγ (pg/ml)	5.92	3.10	6.41	2.48	6.16	3.38	0.8630
IL10 (pg/ml)	9.87	3.69	8.13^*^	2.74	9.08	3.41	0.5265
IL1β (pg/ml)	0.90	0.41	1.15^*^	0.62	1.11^##^	0.75	0.0006
NO (uM)	6.13	5.74	9.54^**^	5.97	8.05	4.29	0.0606

Data are presented as median ± interquartile range (IQR).

^¤^ presented as mean ± standard error of mean (SEM).

^#, *^ p<0.05. Statistically different versus normal weight children by t-test.

^## ,**^ p<0.01. Statistically different versus normal weight children by t-test.

### Altered cytokine profiles in overweight and obese adolescents

Compared with their normal weight counterparts, overweight adolescents had lower serum IL10 but higher TNFα, NO and IL1β concentrations (all p<0.05; Table 2). Obese adolescents had increased IL1β but decreased hepcidin concentration compared with the respective concentration in normal weight adolescents (p<0.01 and p<0.05; respectively). There were no differences in circulating IFNγ between the two groups. No sex differences were found in cytokine profiles except IL1β. Overweight/obese boys and girls had significantly higher serum IL1β concentration compared with respective normal weight boys and girls (boys: 1.19±0.05 v.s. 1.0±0.03 pg/ml; p<0.05; girls: 1.17±0.06 v.s. 0.94±0.03 pg/ml; p<0.01) (data not shown).

We grouped the individuals according to the IL10 concentration to investigate the potential confounding variables that are associated with IL10 concentration. The clinical characteristics of the study adolescents in relation to tertile groups of IL10 are shown in Table 1. An inverse correlation was found between BMI and IL10 tertiles but it did not reach statistical significance (Table 1). Furthermore, no correlation was found between IL10 terriles and the individual components of metabolic syndrome. IL10 tertiles were positively correlated with transferrin saturation (p=0.0167) and serum hepcidin (p<0.0001) but inversely correlated with serum total iron binding capacity (TIBC) (p=0.011). Pro-inflammatory cytokines such as TNFα, IL1β and NO were inversely correlated with IL10 tertiles (all p<0.0001).

### Significant correlation between pro-inflammatory cytokines and IL10 concentration

We next performed correlation analysis to identify the possible variables that were associated with IL10. Pearson’s correlations between log serum IL10 and selected laboratory parameters are shown in Table 3. After adjusting for gender, IL10 was inversely correlated with log serum TIBC (r=−0.137; p=0.013) and positively correlated with log-transferrin saturation (r=0.131; p=0.018) and log-hepcidin (r=0.271; p<0.0001). Furthermore, strong inverse correlations were found between IL10 and IL1β (*r*=−0.328, *P*<0.0001), IL10 and TNFα (*r*=−0.209, *P*=0.0001), IL10 and NO (*r*=−0.28668, *P*<0.0001), and between IL10 and IFNγ (*r*=−0.139, *P*=0.012) (Table 3). We next separated overweight and obese adolescents from normal weight. All cytokines investigated continued to have strong inverse correlations with IL10 in normal weight adolescents [IFNγ (*r*=−0.253), TNFα (*r*=−0.1773), IL1β (*r*=−0.347), NO (*r*=−0.311); all p<0.0001]. However, only IL1β was significantly correlated with IL10 in overweight and obese adolescents (*r*=−0.376, *P*<0.0001) (data not shown).

**Table 3:** Pearson’s rank correlation coefficient and partial r of log-transformed serum IL10 with selected anthropometric, iron status, individual components of MetS and inflammatory indicators analyzed in all subjects (n=325)

	**Model 1**^**a**^		**Model 2**^**b**^	
	**r**	***p *value**	**r**	***p *value**
Gender	−0.03064	0.5821		
Log Body fat (%)	0.04426	0.4265	0.0396	0.4775
Log BMI(kg/m^2^)	−0.06153	0.2687	−0.06149	0.2697
Log waist(cm)	0.08005	0.1499	0.08226	0.1396
Log Sytolic BP(mmHg)	−0.00615	0.9125	−0.00621	0.9113
Log Diastolic BP(mmHg)	0.03219	0.5631	0.02999	0.5906
Log Total cholesterol(mg/dL)	−0.05163	0.3535	−0.05337	0.3383
Log Triglyceride(mg/dL)	−0.04992	0.3697	−0.05166	0.3540
Log HDL cholesterol (mg/dL)	0.04768	0.3915	0.04941	0.9285
Log LDL- cholesterol (mg/dL)	−0.00157	0.9775	−0.00501	0.9285
Log Fasting serum insulin (μIU/ml)	0.02903	0.6021	0.02846	0.6097
Log Fasting glucose (mg/dL)	0.04581	0.4105	0.04758	0.3933
HOMA-IR	0.03339	0.5487	0.03309	0.5528
Log Serum iron(ug/dL)	0.09526	0.0864	0.0955	0.0861
Log Serum TIBC(ug/dL)	−0.13754	0.0131	−0.13789	0.0130
Log Serum ferritin(ng/ml)	−0.02723	0.3535	−0.02548	0.6477
Log Transferrin Saturation	0.13037	0.0131	0.1307	0.0186
Log Hepcidin (ng/ml)	0.26157	<0.0001	0.27054	<0.0001
Log IL1β (pg/ml)	−0.32945	<0.0001	−0.32826	<0.0001
Log TNFα (pg/ml)	−0.21078	0.0001	−0.20954	0.0001
Log IFNγ(pg/ml)	−0.14038	0.0113	−0.13892	0.0123
Log NO (μM)	−0.285	<0.0001	−0.28661	<0.0001
Log GOT (IU/L)	0.03548	0.5239	0.04253	0.4455
Log GPT (IU/L)	−0.09192	0.0981	−0.08804	0.1137

^a^ Model1: Crude.

^b^ Model2: Adjusted for gender.

### IL1β Is independently correlated with IL10 concentration

We next performed multiple linear regression analysis to predict variants that were independently associated with IL10 concentration. Thus, serum IL10 was entered as dependent variable. The independent covariates included anthropometry indices (% body fat, BMI, waist circumference), blood glucose and lipid profile (SBP/DBP, total cholesterol, TG, HDL, LDL, fasting insulin, fasting glucose, HOMA-IR index), iron parameters (serum iron, serum TIBC, serum ferritin, transferrin saturation, hepcidin) and pro-inflammatory cytokines (IL1β, TNFα, IFNγ, NO). The initial linear regression analysis showed no correlation between IL10 concentration and anthropometry indices, fasting blood glucose, and lipid profiles. Therefore, only iron parameters (serum iron, serum TIBC, serum ferritin, transferrin saturation, hepcidin) and pro-inflammatory cytokines (IL1β, TNFα, IFNγ, NO) were selected for multivariate analyses.

**Figure 1:**
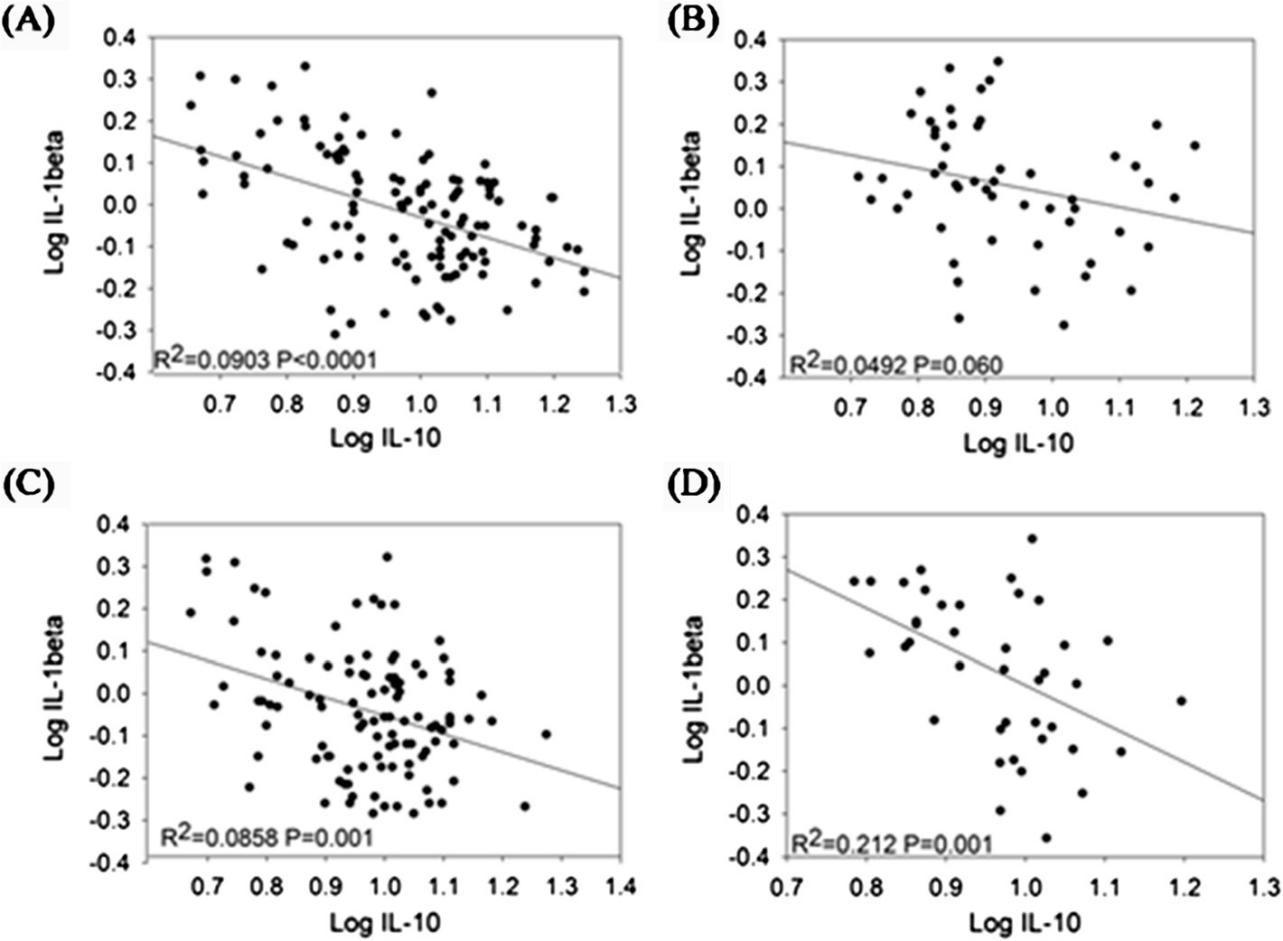
**Correlation between IL10 and IL1β stratified by gender and BMI in 340 subjects (182 boys and 158 girls).** Pearson’s correlation coefficient correlation between IL10 and IL1β in normal weight boys **(A)**, overweight/obese boys **(B)**, normal weight girls **(C)** and **(D)** overweight/obese girls.

IL1β was independently associated with IL10 (β=−0.157, p<0.0001), after adjusting for gender, serum TIBC, transferrin saturation, NO, TNFα, IFNγ and NO (Table 4; pooled, Multivariate model: B). When overweight/obese adolescents were assessed separately from normal weight, only IL1β was inversely associated with serum IL10 (β=−0.231, p=0.0009) (Table 4; overweight and obese, Model D). The association between IL10 and IL1β became weaker in adolescents with normal weight (β=−0.157, p=0.0002), after further controlling for gender, NO, TNFα, IFNγ and NO (Table 4; pooled, Multivariate model: C). The relationship between IL10 and IL1β appears to differ in the normal weight and overweight/obese children. All students were from the first year junior high school, so there was very little variation in age. The correlation between IL10 and IL1β was similar between girls and boys (R^2^=0.110 v.s. R^2^=0.0908) and between overweight/obese and normal weight (R^2^=0.113 v.s. R^2^=0.0912). However, the correlation between IL10 and IL1β in normal weight girls differed from those of overweight/obese girls (R^2^=0.0858 v.s. R^2^=0.212; respectively) and between normal weight and overweight/obese boys (R^2^=0.0903 v.s. R^2^=0.0492; respectively) (Figure 1). These data indicate that IL1β is independently correlated with IL10 homeostasis and this association is more pronounced in girls who are overweight or obese.

**Table 4:** Linear regression analyses of predictors of serum IL10 in young adolescents

	**Crude**		Model A^*^		**Multivariate model (B)**^**#**^	
**Pooled (n=325)**	**β**	***p-value***	**β**	***p-value***	**β**	***p-value***
Log Serum iron(ug/dL)	0.06702	0.0864	0.06716	0.0861		
Log Serum TIBC(ug/dL)	−0.30981	0.0131	−0.31047	0.013	−0.1578	0.2026
Log Serum ferritin(ng/ml)	−0.01122	0.6248	−0.01051	0.6477		
Log Transferrin Saturation (%)	0.08652	0.0187	0.08670	0.0186	0.04459	0.21111
Log Hepcidin (ng/ml)	−0.1275	0.0478	−0.13164	0.0502		
Log IL1β (pg/ml)	−0.21868	<0.0001	−0.21834	<0.0001	−0.1568	<0.0001
Log TNF (pg/ml)	−0.15167	0.0001	−0.15092	0.0001	−0.08206	0.0317
Log IFNγ (pg/ml)	−0.12521	0.0113	−0.12405	0.0123	−0.11378	0.0124
Log NO (μM)	−0.12417	<0.0001	−0.12491	<0.0001	−0.08628	0.0002
**Normal weight (n=231)**	**Crude**		Model A^*^		**Multivariate model (C)**^※^	
	**β**	***p-value***	**β**	***p-value***	**β**	***p-value***
Log Serum iron(ug/dL)	0.05598	0.2483	0.05604	0.2487		
Log Serum TIBC(ug/dL)	−0.29762	0.0665	−0.29704	0.0676		
Log Serum ferritin(ng/ml)	−0.02808	0.3080	−0.02826	0.3061		
Log Transferrin Saturation (%)	0.07590	0.1022	0.07590	0.1029		
Log Hepcidin (ng/ml)	−0.19145	0.3327	−0.23717	0.2584		
Log IL1β (pg/ml)	−0.20687	<0.0001	−0.20810	<0.0001	−0.1572	0.0002
Log TNF (pg/ml)	−0.16167	0.0007	−0.16165	0.0008	−0.0829	0.07
Log IFNγ (pg/ml)	−0.13909	0.0178	−0.13939	0.0178	−0.10408	0.0548
Log NO (μM)	−0.14744	<0.0001	−0.14735	<0.0001	−0.10764	0.0002
**Overweight and obese (n=94)**	**Crude**		Model A^*^		**Model D**^**§**^	
	**β**	***p-value***	**β**	***p-value***	**β**	***p-value***
Log Serum iron(ug/dL)	0.08013	0.2147				
Log Serum TIBC(ug/dL)	−0.31443	0.0932				
Log Serum ferritin(ng/ml)	0.03041	0.4575	0.04130	0.3155		
Log Transferrin Saturation (%)	0.09707	0.0975	0.10025	0.0848		
Log Hepcidin (ng/ml)	−0.08019	0.2256	−0.06115	0.3845		
Log IL1β (pg/ml)	−0.23952	0.0005	−0.23111	0.0009	−0.23111	0.0009
Log TNF (pg/ml)	−0.09581	0.1846	−0.07848	0.2831		
Log IFNγ (pg/ml)	−0.07007	0.4424	−0.04873	0.5962		
Log NO (μM)	−0.04679	0.2784	−0.05602	0.1944		

^*^Model A: Adjusted for gender.

^#^Model B: Multivariate model adding gender.

^※^Model C: Multivariate model adding gender.

^§^Model D: Adjusted for gender.

## Discussion

In the present study, we confirmed that young adolescents who are overweight and obese had decreased circulating IL10 concentration and increased pro-inflammatory cytokines TNFα, IL1β and NO. Our study also found that pro-inflammatory cytokine IL1β and IL10 are independently associated. A decline in serum IL10 concentration in overweight and obese adolescents may further contribute to the IL1β-mediated inflammatory environment associated with obesity. IL1β has been associated with the destruction of the insulin-producing beta cell [28]. Administration of neutralizing monoclonal antibodies to IL1β improved glycemic control and beta cell function in type 2 diabetic patients [29]. Our study showed IL10 concentration was neither correlated with blood glucose homeostasis variables nor blood lipid profiles. This is partially explained by the low prevalence rate of MetS in the study subjects. IL10 plays a critical role in limiting inflammation and a switch in anti- and pro-inflammatory balance towards pro-inflammatory state in overweight and obese adolescents may promote the progression of normal glucose tolerance to insulin resistance in adulthood [30]. The pathogenesis of type 2 diabetes is complex, involving the interaction of genetic and environmental risk factors. Therefore, a longitudinal follow up study on obese children and adolescents is needed to clarify the role of IL10 in the progression of type 2 diabetes and MetS.

The association between IL10 and IL1β was more pronounced in girls who are overweight or obese. Both overweight/obese boys and girls had elevated serum IL1β concentrations. However, correlations between IL10 and IL1β concentrations were greater in overweight/obese girls (R^2^=0.212) than boys (R^2^=0.049). Corcoran *et al.* investigated the effect of estrogen and testosterone on the expression of pro-inflammatory mediators in macrophages obtained from patients with coronary heart disease [31]. The authors showed testosterone reduced the expression of TNFα and IL1β; by contrast, estrogen did not have these adverse effects [31]. In our study, serum IL1β concentrations were similarly increased in both overweight/obese boys and girls. This suggests that the difference between the two groups is less likely to be explained by the effect of sex hormones on IL1β. Adipose tissues derived adiponectin is a potential mediator of IL10 [32]. Adiponectin concentrations are inversely correlated with waist circumference, BMI and total body fat [33]. We found that overweight/obese boys had slightly higher waist circumference than overweight/obese girls (84.7±3.2 cm v.s. 81.1±2.7 cm; p=0.06). Although it did not reach statistical significance, overweight/obese boys had slightly lower IL10 concentrations when compared with overweight/obese girls. This contributed to a lower ratio of IL10/ IL1β in overweight/obese boys than girls (8.77±0.65 v.s. 9.93±0.86; p=0.35). We hypothesize that adiponectin concentrations may contribute to the gender difference in IL10 and IL1β association in overweight/obese adolescents.

Interleukins (ILs) are key mediators of the innate immune response and inflammatory process. So far, 11 members of the IL1 family have been identified including IL1α, IL1β and IL1 receptor antagonist (IL1Ra) [28]. Circulating IL1β and IL1Ra are elevated in patients with obesity and type 2 diabetes [34]. Short-term IL1α treatment transiently causes insulin resistance at insulin receptor substrate 1 level in 3T3L1 adipocytes [35]. IL1Ra is regarded as anti-inflammatory cytokine but it does not directly elicit an anti-inflammatory response. It binds tightly to IL1α and IL1β receptor on the cell surface, hence blocking the activity of either IL1α or IL1β. A recent report showed 13 weeks of IL1Ra therapy improved glycemic control and the function of the insulin-producing beta cell in patients with type 2 diabetes [34].

In this study, we also observed a positive relationship between IL10 and iron status. Associations between obesity and poor iron status have recently been described in White and Hispanic children [36,37]. However, we found no difference in iron status between normal body weight and overweight/obese adolescents (data not shown). The relationship between IL10 and iron metabolism is poorly understood. Patients with Crohn’s disease receiving higher doses of IL10 developed anemia and a dose-dependent increase of serum ferritin concentration [19]. The authors suggested that hyperferritinemia may result from direct stimulation of ferritin translation by IL10 via the suppression of the binding affinity of iron regulatory proteins to the 5’-untranslated region of ferritin mRNA in human monocytic cells [19]. Macrophages play a key role in the iron homeostasis. Through erythrophagocytosis of senescent red blood cells, macrophages recycle and redistribute heme iron to the plasma. Chau *et al.* showed IL10 triggers expression of heme oxygenase 1 (HO-1) via the p38 MAPK pathway and IL10-HO1 pathways are critical for the protection against LPS-induced septic shock in mice [38]. These data suggest that abnormal high concentration of IL10 may limit iron bioavailability to erythroid progenitor cells; whilst, defective IL10 production may impair HO1 mediated anti-inflammatory and anti-oxidant responses. Nevertheless, the consequences of defective IL10 synthesis and erythrogenesis in overweight and obese adolescents need to be further investigated in a longitudinal study.

There are several limitations in our study. The cross-sectional nature of the current study and the relative small sample size are one of the limitations. Further, the inverse correlation between serum IL10 and pro-inflammatory cytokines may be casual and related to IL10 polymorphisms. IL-10 secretion is tightly controlled and under a stringent genetic regulation with 75% of heritability [39]. The three polymorphisms -1082G/A, -819C/T, and -592C/A in the IL10 promoter region were reported to influence IL10 transcription. A promoter polymorphism (−592C/A) was associated with lower circulating IL-10 levels and an increased risk for obesity and insulin resistance in Italian people [40]. However, this association was not confirmed in the Chinese population [41]. Another limitation is that we could not confirm which IL10-producing cells are affected in obese children.

## Conclusions

In conclusion, given the increasing numbers of obese children in the population, it is critical to investigate the factors associated with the obesity-related declines in health. Our study confirmed that low IL10 concentration is associated with overweight and obesity in young adolescents. A decline in serum IL10 concentration in overweight and obese adolescents may further contribute to the IL1β-mediated inflammatory environment associated with obesity.

## Abbreviations

IL10: Interleukin 10; NO: Nitric oxide; MetS: Metabolic syndrome; SF: Serum ferritin; TS: Transferrin saturation; TIBC: Total iron binding capacity; BMI: Body mass index; LDL: Low density lipoprotein cholesterol; HDL: High density lipoprotein cholesterol; TG: Triglyceride; SEM: Standard error of mean; PBMC: Peripheral blood mononuclear cells; HO-1: Heme oxygenase 1; LPS: Lipopolysaccharide; FPG: Fasting plasma glucose concentration; IL1Ra: IL1 receptor antagonist.

## Competing interests

All authors declare that they have not received support (financial and nonfinancial) from any companies for the submitted work; no authors have any relationship with companies that may have an interest in the submitted work; their spouses, partners, or children.

## Authors’ contributions

JSC conceptualized and designed the study, drafted the initial manuscript, and approved the final manuscript CCC and EYC contributed to the study design, data acquisition and initial data analysis. SSL conducted preparation and examination of cytokines. CST carried out the iron biochemistry analysis and CHB supervised the data collection and statistical analysis. KCC critically reviewed the manuscript and approved the final manuscript. All authors read and approved the final manuscript.

## Pre-publication history

The pre-publication history for this paper can be accessed here:

http://www.biomedcentral.com/1471-2431/13/123/prepub
